# HLA-G 14bp Ins/Del Polymorphism in the 3′UTR Region and Acute Rejection in Kidney Transplant Recipients: An Updated Meta-Analysis

**DOI:** 10.3390/medicina57101007

**Published:** 2021-09-24

**Authors:** Sang Wook Kang, Eunkyung Oh, Wonwoo Cho, Minseok Kim, Eo Jin Park, Kyu Hwan Kwack, Kang Chung, Ok Hyung Nam, Yong Kwon Chae, Ju Yeon Ban

**Affiliations:** 1 Department of Oral and Maxillofacial Pathology, School of Dentistry, Kyung Hee University, Seoul 02453, Korea; pathemis@khu.ac.kr; 2 Department of Medicine, Graduate School, Kyung Hee University, Seoul 02453, Korea; goblinhat@khu.ac.kr (E.O.); biochemi@khu.ac.kr (W.C.); mskim9262@khu.ac.kr (M.K.); 3 Department of Physical Medicine and Rehabilitation, Graduate School, Kyung Hee University, Seoul 02453, Korea; cp1024@naver.com; 4 Department of Oral Biology, University at Buffalo, Buffalo, NY 14214, USA; dmdkwack@gmail.com; 5 Department of Oral Anatomy and Developmental Biology, Graduate School of Dentistry, Kyung Hee University, Seoul 02453, Korea; dentallica@naver.com; 6 Department of Pediatric Dentistry, School of Dentistry, Kyung Hee University, Seoul 02453, Korea; pedokhyung@gmail.com (O.H.N.); pedochae@gmail.com (Y.K.C.); 7 Department of Dental Pharmacology, School of Dentistry, Dankook University, Cheonan 16890, Korea

**Keywords:** human leukocyte antigen G, HLA-G, polymorphism, acute kidney injury, acute rejection, meta-analysis

## Abstract

*Background and Objectives:* Acute kidney injury (AKI) affects the survival rate of kidney transplant organs and patients. Acute rejection (AR) due to AKI may lead to kidney transplantation failure. It is known that there is a relationship between human leukocyte antigen-G (HLA-G), which is involved in immune regulation, and AR in transplant patients. Moreover, 14-bp insertion/deletion polymorphism in the 3′ untranslated region (UTR) region of the HLA-G gene is known to affect HLA-G expression. However, its relationship to AR is still controversial. The aim of this study was to investigate whether HLA-G 14-bp insertion/deletion polymorphism contributed to the development of AR in kidney transplant patients using a meta-analysis. *Materials and Methods:* To perform our meta-analysis, eligible studies about HLA-G 14-bp insertion/deletion polymorphism and AR were searched in electronic databases until 1 June 2021. Finally, a total of 336 patients with AR and 952 patients without AR in relation to kidney transplantation were analyzed from a total of nine studies. *Results:* In our results, the Del allele and Ins/Del+Del/Del and Del/Del genotypes significantly increased susceptibility of AR in Asian populations [odds ratio (OR) = 2.359, 95% confidence interval (CI) = 1.568–3.550, *p* = 3.8 × 10^−5^; OR = 3.357, 95% CI = 1.769–6.370, *p* = 0.002; OR = 2.750, 95% CI = 1.354–5.587, *p* = 0.0052 in each model, respectively]. *Conclusions:* Evidence of the present results indicate that HLA-G 14-bp insertion/deletion polymorphism is associated with susceptibility to AR in the Asian population.

## 1. Introduction

The World Health Organization (WHO) defines health as “A state of complete physical, mental, and social well-being and not merely the absence of disease or infirmity” [1]. In the field of healthcare, the treatment of diseases and the importance of quality of life have both increased [2]. Acute kidney injury (AKI) ranges from minor to fatal damage of the kidney. Decreased kidney function due to AKI leads to fluid retention, anemia, impaired bone and mineral metabolism, dyslipidemia, and malnutrition [3]. If treatment is not performed properly, a kidney transplantation or dialysis may be required to replace kidney function. This condition is known as end-stage renal disease (ESRD). ESRD has a poor prognosis and can lead to death if not properly treated [4]. Treatment of ESRD includes hemodialysis, peritoneal dialysis, and kidney transplantation [5]. Among these, kidney transplantation and immunosuppressive therapy can be a good treatment choice. With this treatment method, renal function was restored in patients with ESRD, despite AKI, and mortality was reduced [6].

Unfortunately, AKI is often observed after kidney transplantation. AKI can develop from medication, bacterial infection, urinary tract obstruction, kidney disease, immunologic factors, etc. Among them, AR is related to immunologic factors [7]. AR can lead to graft loss and kidney transplantation failure [8].

HLA-G is a non-classical human leukocyte antigen G class I molecule. It was initially known as a molecule that protected the fetus from the immune system of its mother [9,10]. HLA-G plays a role in inhibiting or regulating maternal/fetal decidual macrophages by changing the behavior of antigen-presenting cells or cytokine production through binding to immunoglobulin-like transcript-2 (ILT-2) and ILT-4 receptors. It also inhibits natural killer (NK) cells through binding with killer cell immunoglobulin like receptor, two Ig domains and long cytoplasmic tail 4 (KIR2DL4) [11].

As HLA-G plays an important role in immunosuppression, it has a close relationship with the success of heart or kidney transplantation [12,13]. A previous study reported that HLA-G might be related to the development of AR [14]. Among polymorphisms in HLA-G, 14-bp insertion/deletion alters HLA-G function by affecting mRNA expression in the 3’ untranslated region (3’UTR) [15]. Therefore, several studies were performed to elucidate the relationship between HLA-G 14-bp insertion/deletion polymorphism and AR during transplantation [16,17,18,19,20,21,22,23,24]. The results were split into two directions, namely the polymorphism of HLA-G was related or was not related to the development of AR. These conflicting results are attributable to the different races, study sizes, and sample powers of the cohorts. A meta-analysis could provide statistical significance for these controversial results.

The purpose of this study is to elicit a more accurate conclusion by performing an updated meta-analysis to obtain more reliable results regarding the relationship between HLA-G 14-bp insertion/deletion polymorphism and AR.

## 2. Materials and Methods

### 2.1. Search Strategy

**Figure 1:**
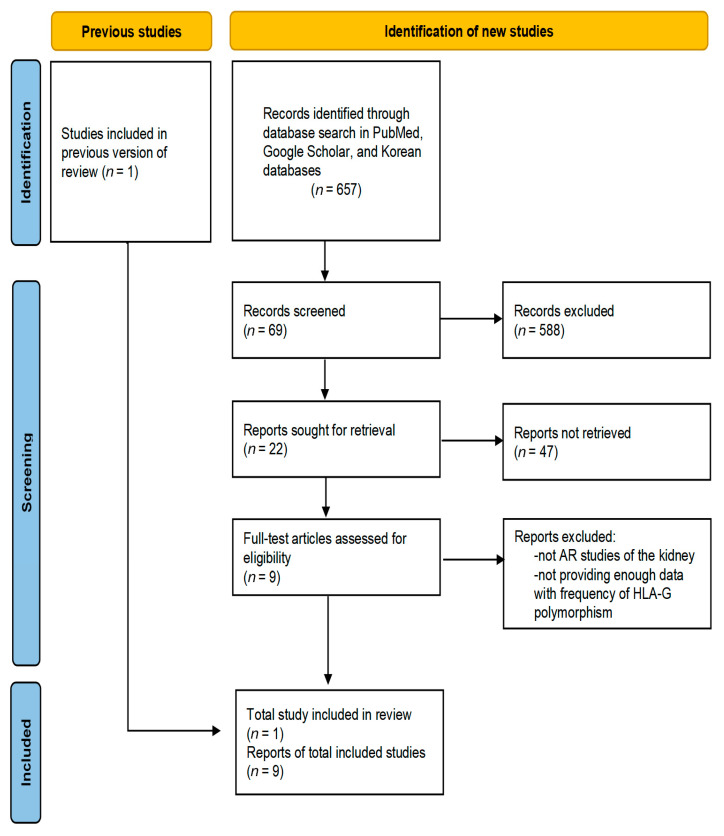
PRISMA flowchart in present study.

By 1 June 2021, a comprehensive electronic search was performed, including on PubMed, Google Scholar, and Korean databases, for all relevant studies reporting a relationship between HLA-G polymorphism and the development of AR. “Human leukocyte antigen G”, “HLA-G”, “polymorphism”, “variant”, “rs16375”, “14bp”, “acute rejection”, or “kidney” to find candidate papers for meta-analysis in various database, and “meta-analysis” was used to find related papers. Previous meta-analysis studies about HLA-G and graft survival in kidney transplant recipients were considered as reference (Figure 1).

### 2.2. Study Selection

Among the candidate papers, a case contro l study design and a study of the association between acute rejection and insertion/deletion of polymorphism in the HLA-G gene were selected for our final meta-analysis. Among the selected papers, those deviating from the Hardy-Weinberg equilibrium (HWE) test were excluded from the meta-analysis.

### 2.3. Data Extraction

To provide information from the papers selected for the final meta-analysis, the name of the main author and publication year were extracted. For meta-analysis, statistics, race, acute rejection patients, number of controls, and genotype of HLA-G insertion (Ins)/deletion (Del) polymorphism frequencies were extracted for each group.

### 2.4. Statistical Analysis

To evaluate whether there was heterogeneity in the data extracted from the papers for meta-analysis, Q test and I^2^ statistics were performed. A fixed effect model or a random effect model was considered according to the Q test and I^2^ statistics values. Next, the Hardy-Weinberg equilibrium (HWE) in the control group was calculated from the extracted data. Egger’s test and sensitivity analysis were performed for publication bias and impact of eligible studies. The relationship between the polymorphism of the HLA-G gene and AR was analyzed using the data, confirming that there was no error in the Hardy-Weinberg equilibrium. The allele, recessive, dominant, and overdominant models were applied for genetic statistical models. For statistical significance, the *p* value was used. The *p* < 0.05 was regarded as a statistically significant association with acute rejection in kidney transplantation patients. Meta-analysis was performed using comprehensive meta-analysis software (https://metagenyo.genyo.es, accessed on 1 September 2021).

## 3. Results

The eligible studies included in our updated meta-analysis are summarized in Table 1. Briefly, we performed an updated meta-analysis with nine studies [12,13,14,15,16,17,18,19,20]. After pooling all data, these 9 studies, including 336 patients with acute rejection and 952 patients with no AR in kidney transplant recipients, were analyzed in our meta-analysis.

Among these nine studies, none showed an error in the HWE in the control group (*p* > 0.05, Table 1). To estimate the potential risk in all AR cases and non-AR cases, heterogeneity tests were performed for different analysis models, respectively (dominant, recessive, overdominant, and allele model). The results of the heterogeneity test for our meta-analysis are shown in Table 2 and Table 3. If the result of the Q test was *p* < 0.05 or the I^2^ statistic was >50%, a random-effects method was adopted. Otherwise, a fixed-effects method was applied.

**Figure 2:**
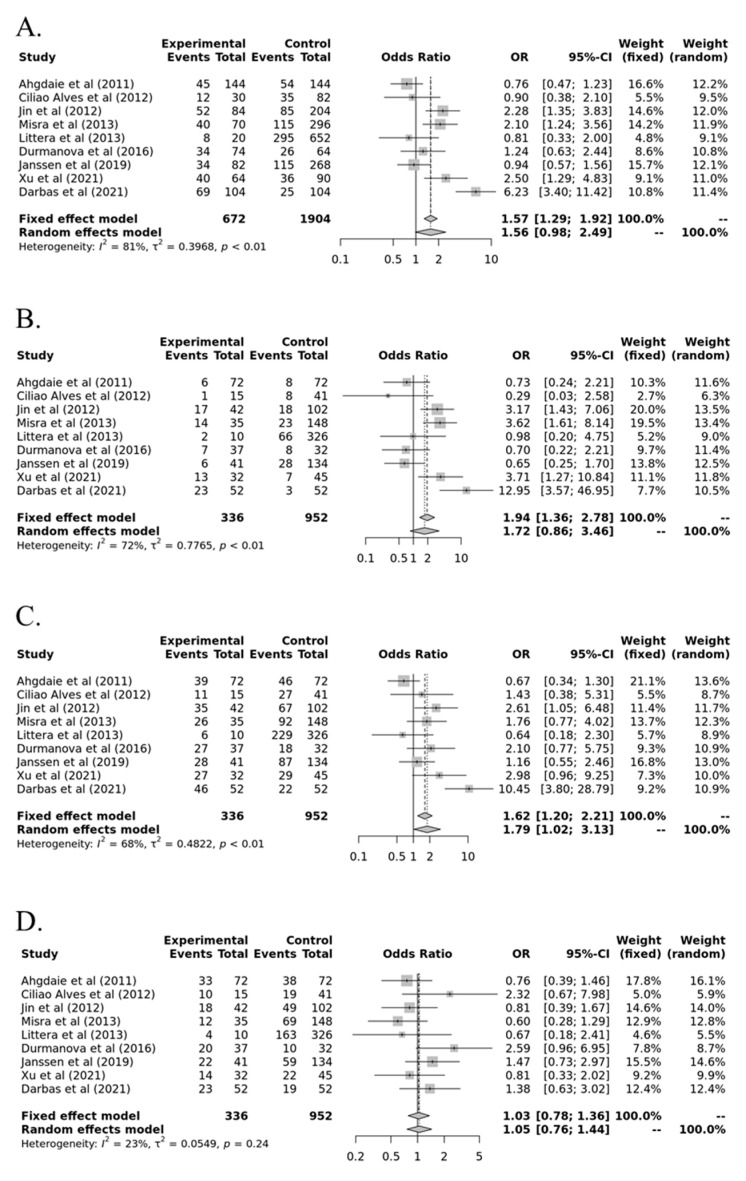
Forest plots in genetic models between HLA-G polymorphism (14-bp insertion/deletion (*rs16375*)) and susceptibility to acute rejection in kidney transplant recipients. (**A**) Ins vs. Del; (**B**) Ins/Ins vs. Ins/Del+Del/Del; (**C**) Ins/Ins+Ins/Del vs. Del/Del; (**D**) Ins/Del vs. Ins/Ins+Del/Del.

**Figure 3:**
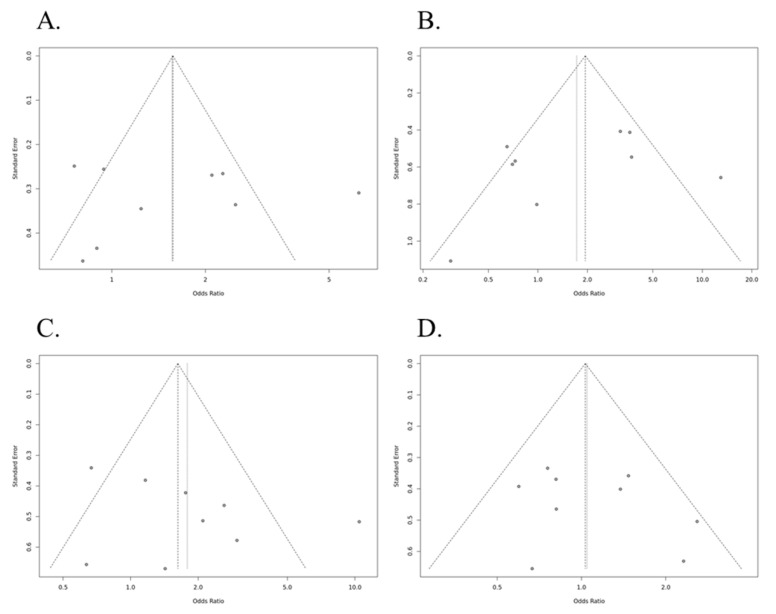
Publication bias in genetic models between HLA-G polymorphism (14-bp insertion/deletion (rs16375)) and susceptibility to acute rejection in kidney transplant recipients. (**A**) Ins vs. Del; (**B**) Ins/Ins vs. Ins/Del+Del/Del; (**C**) Ins/Ins+Ins/Del vs. Del/Del; (**D**) Ins/Del vs. Ins/Ins+Del/Del.

In an allele analysis (Ins allele vs. Del allele) between HLA-G and susceptibility for AR, the minor Del allele showed no association with AR [all populations, odds ratio (OR) = 1564, 95% confidence interval (CI) = 0.983–2.486, *p* = 0.411 in random model] (Table 2 and Figure 2). Moreover, no significant association was observed between each genotype and risk of AR in recessive (Ins/Ins vs. Ins/Del+Del/Del, all population, OR = 1.721, 95% CI = 0.856–3.462, *p* = 0.895 in random model), dominant (Ins/Ins+ Ins/Del vs. Del/Del, OR = 1.790, 95% CI = 1.023–3.131, *p* = 0.290 in random model), or overdominant (Ins/Del vs. Ins/Ins+Del/Del, OR = 1.032, 95% CI = 0.783–1.361, *p* = 1.000 in fixed model) models, respectively (Table 2 and Figure 2). As shown in Figure 3, no publication bias was observed (*p* > 0.05).

**Figure 4:**
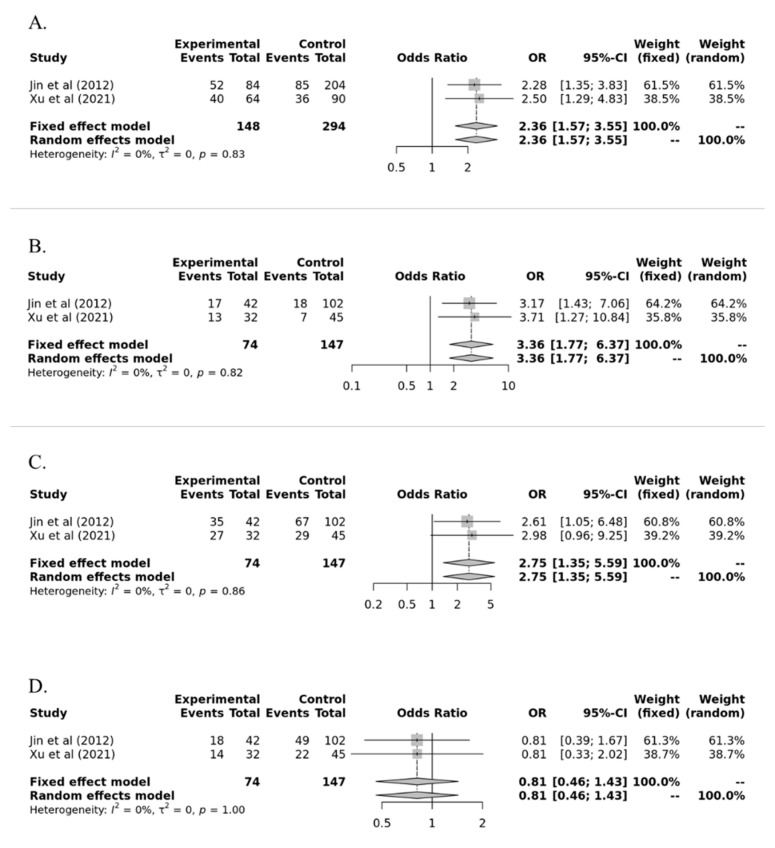
Subgroup analysis between HLA-G polymorphism (14-bp insertion/deletion (rs16375)) and susceptibility to acute rejection in kidney transplant recipients according to nationality. (**A**) Ins vs. Del; (**B**) Ins/Ins vs. Ins/Del+Del/Del; (**C**) Ins/Ins+Ins/Del vs. Del/Del; (**D**) Ins/Del vs. Ins/Ins+Del/Del.

We also performed a subgroup analysis according to nationality. Table 3 presents the pooled results of our subgroup analysis between HLA-G polymorphism (Ins/Del) and susceptibility to AR in Caucasian and Asian populations. The Del allele in allele model and Ins/Del+Del/Del and Del/Del genotypes significantly increased susceptibility to AR in the Asian population (OR = 2.359, 95% CI = 1.568–3.550, *p* = 3.8 × 10^−5^; OR = 3.357, 95% CI = 1.769–6.370, *p* = 0.002; OR = 2.750, 95% CI = 1.354–5.587, *p* = 0.0052, respectively) (Table 3 and Figure 4).

The results obtained from these genetic models suggested that HLA-G polymorphism (Ins/Del) was significantly associated with a strong risk of AR in the Asian population.

## 4. Discussion

In a meta-analysis published in 2012, it was reported that the HLA-G 14-bp Ins/Del polymorphism was not associated with AR [25]. Our meta-analysis showed that the HLA-G 14-bp Ins/Del polymorphism was not associated with AR in all races, but it was, however, linked with AR in Asians. Our findings contrast with the previous study describing no association. This difference seems to be due to an incorrectly performed meta-analysis. The other study’s meta-analysis used Piancatelli’s data inappropriately, and the Littera and Cilião Alves results were used without excluding chronic rejection; thus, they included both chronic rejection and AR. Therefore, we performed an updated meta-analysis by excluding incorrect data and adding new studies. Our meta-analysis results showed no significance when both Caucasians and Asians were included but showed a strong association with AR in the Asian population. The Del allele and genotype included Del allele, which increased the susceptibility to AR in allele, recessive, and dominant models. According to the 1000 Genomes Project, the Ins/Del allele frequencies in all populations—American, European, African, South Asian, and East Asian—have been reported to be 0.394 and 0.606, 0.385 and 0.615, 0.366 and 0.634, 0.382 and 0.618, 0.525 and 0.475, and 0.318 and 0.682, respectively [26]. Allele frequencies vary among races, with higher rates of Del allele in East Asians and lower rates of Del allele in South Asians. Previous studies reported that the success rate of kidney transplantation differs by race, and the incidence of AR in heart transplantation also varies by race [27,28].

NK cells can be inhibited by HLA-G. The NK cell subset plays an important role in the development of AR. A previous study reported that the ratio of NK cells with CD3-CD56 bright subsets increased in the case of patients with AR [29]. NK cells are involved not only in AR, but also in tolerance induction by regulating immune response [30]. In addition, HLA-G is involved in immune regulation by reducing CD8 T-cell cytotoxicity and CD4 T-cell expansion. Thereby, HLA-G improves acceptance in transplant patients, and expression of HLA-G reduces the development of AR [10].

HLA-G 14-bp Ins/Del polymorphism was the first reported polymorphism in HLA-G [31]. HLA-G 14-bp Ins/Del polymorphism is involved in splicing and affects mRNA stability and soluble HLA-G level [32,33]. Soluble HLA-G levels are known to be related to the success of kidney transplantation or the development of AR. Soluble HLA-G levels are increased earlier and remain higher in patients without rejection but not in patients with AR [34]. HLA-G is known to be able to inhibit alloproliferative responses [35]. A previous study reported that soluble HLA-G level was increased after immunosuppressive therapy in patients with heart transplantation [13]. As HLA-G 14-bp Ins/Del polymorphism is associated with mRNA stability and soluble HLA-G level, the polymorphism has been studied extensively in various diseases such as diabetes mellitus, Crohn’s disease, cancer, and celiac disease [32,33,34,35,36,37]. The Ins allele increases mRNA stability and decreases HLA-G expression while the Del allele increases [38]. After allogeneic stem cell transplantation, progression-free survival and overall survival are the longest in patients with the Del allele [39]. In addition, HLA-G 14-bp Ins/Del polymorphism is associated with a response to immunosuppressive therapy. In the Del/Del genotype, the highest soluble HLA-G levels were observed after methotrexate treatment [40]. As mentioned above, Del allele frequency is the highest in healthy East Asians. As HLA-G plays a role in immunosuppression, this difference in frequency seems to cause the lowest development of AR and the highest transplantation success rate among races [27,28]. In our meta-analysis, the high frequency of the Ins allele in the Asian acute rejection group seems to be consistent with these results.

Several previous studies investigated the relationship between HLA-G 14-bp Ins/Del polymorphism and AR and showed contradictory results. Some studies have reported an association between HLA-G 14-bp Ins/Del polymorphism and AR, while others have reported no association. In this study, we collected eligible studies, organized data by reviewing the results of previous studies, and performed a meta-analysis. The genetic distribution of HLA-G 14-bp Ins/Del polymorphism is known to be different between races, and our study showed that there is genetic variation and different results in the meta-analysis when considering race [41]. According to a previous study, it was reported that the results of a kidney allograft differ between races, which may be due to genetic diversity [27]. However, this is also a limitation of our meta-analysis. Due to the insufficient number of studies, we had no choice but to divide the subgroups into only Caucasian and Asian. In addition, it was difficult to identify monoethnicity or multi-raciality. The reason that statistically significant results could be observed only in the Asian population is thought to be because the patients in the two studies included in the meta-analysis were recruited from the same region. In addition, as shown in Table 1, the participating patients were treated with various immunosuppressive drugs, even in the same study. Additionally, the type of acute rejection, whether antibody-mediated or T-cell mediated, was not considered.

ESRD is a fatal disease, but kidney transplantation could help patients live longer and improve quality of life [4,6]. In general, before kidney transplantation, HLA matching should be performed to prevent AR [42]. Our findings showed that HLA-G 14-bp Ins/Del polymorphism is associated with AR in the Asian population. Checking the HLA-G genotype, in addition to HLA matching, before kidney transplantation might help prevent AR due to AKI and might also improve prognosis.

## 5. Conclusions

In conclusion, we evaluated the relationship between HLA-G 14-bp Ins/Del polymorphism and AR using a meta-analysis. A statistically significant association was detected in the Asian population. If more results for various populations are accumulated in future studies, the relationship between HLA-G 14-bp Ins/Del polymorphism and AR might be revised, and it could help to improve clinical prognosis.

## Figures and Tables

**Table 1:** Characteristics of eligible studies included in the meta-analysis.

Author	Nationality	Immunosuppressive Treatment	Acute Rejection			Non-Acute Rejection			HWE *p*
(Publish Year)			Ins/Ins	Ins/Del	Del/Del	Ins/Ins	Ins/Del	Del/Del	
Ahgdaie et al. (2011)	Iran	cyclosporine, azathioprine, prednisone, mycophenolate mofetil	6	33	33	8	38	26	0.86
Ciliao Alves et al. (2012)	Brazil	(cyclosporine or tacrolimus), methylprednisone, and mycophenolate mofetil	1	10	4	8	19	14	1.00
Jin et al. (2012)	China	(tacrolimus or cyclosporine), mycophenolate mofetil and prednisone	17	18	7	18	49	35	1.00
Misra et al. (2013)	India	tacrolimus, mycophenolate mofetil, prednisolone, cyclosporine, everolimus, basiliximab, antithymocyte globulin	14	12	9	23	69	56	1.00
Littera et al. (2013)	Italy	rapamycin, steroids, and cyclosporine and/or mycophenolate mofetil	2	4	4	66	163	97	1.00
Durmanova et al. (2019)	Slovakia	basiliximab/daclizumab, tacrolimus, mycophenolate mofetil, corticosteroid, cyclosporine, antithymocyte globulin	7	20	10	8	10	14	0.42
Janssen et al. (2019)	Germany	calcineurin inhibitors, (mycophenolate mofetil or mTOR inhibitor) and steroids	6	22	13	28	59	47	0.86
Xu et al. (2021)	China	tacrolimus, mycophenolate mofetil, prednisone, cyclosporine	13	14	5	7	22	16	1.00
Darbas et al. (2021)	Turkey	antithymocyte globulin, basiliximab, mycophenolate mofetil, sirolimus, tacrolimus, everolimus, cyclosporine	23	23	6	3	19	30	1.00

HWE: Hardy-Weinberg equilibrium.

**Table 2:** Overall analysis between HLA-G polymorphism (14-bp insertion/deletion (rs16375)) and susceptibility to acute rejection in kidney transplant recipients.

Genetic Models	Association Test			Heterogeneity		Model	Publication Bias
	OR	95% CI	*p*	*p*	*I* ^2^		Egger’s Test *p*-Val
Allele (Ins vs. Del)	1.564	0.983–2.486	0.411	<0.01	0.81	Random	0.964
Recessive (Ins/Ins vs. Ins/Del+Del/Del)	1.721	0.856–3.462	0.895	<0.01	0.72	Random	0.351
Dominant (Ins/Ins+ Ins/Del vs. Del/Del)	1.790	1.023–3.131	0.290	<0.01	0.68	Random	0.306
Overdominant (Ins/Del vs. Ins/Ins+Del/Del)	1.032	0.783–1.361	1.000	0.23	0.24	Fixed	0.392

OR: odds ratio; CI: confidence interval.

**Table 3:** Subgroup analysis between HLA-G polymorphism (14-bp insertion/deletion (rs16375)) and susceptibility to acute rejection in kidney transplant recipients according to nationality.

Genetic Models	Nationality	Association Test			Heterogeneity		Model	Publication Bias
		OR	95% CI	*p*	*p*	*I* ^2^		Egger’s Test *p*-Val
Allele	Asian	2.359	1.568–3.550	**3.8 × 10^−5^**	0.83	0.00	Fixed	NA
	Caucasian	1.377	0.773–2.453	0.278	<0.01	0.84	Random	0.9832
Recessive	Asian	3.357	1.769–6.370	**0.0002**	0.82	0.00	Fixed	NA
	Caucasian	1.347	0.5456–3.3237	0.518	<0.01	0.75	Random	0.5363
Dominant	Asian	2.750	1.354–5.587	**0.0052**	0.86	0.00	Fixed	NA
	Caucasian	1.590	0.804–3.144	0.183	<0.01	0.73	Random	0.4594
Overdominant	Asian	0.812	0.461–1.431	0.471	1.00	0.00	Fixed	NA
	Caucasian	1.112	0.810–1.527	0.511	0.15	0.37	Fixed	0.4574

OR: odds ratio; CI: confidence interval; NA: not applicable. Bold numbers indicate significant association with risk of AR.

## Data Availability

Not applicable.
